# Livestock water and land productivity in Kenya and their implications for future resource use

**DOI:** 10.1016/j.heliyon.2022.e09006

**Published:** 2022-02-25

**Authors:** Caroline K. Bosire, Nadhem Mtimet, Dolapo Enahoro, Joseph O. Ogutu, Maarten S. Krol, Jan de Leeuw, Nicholas Ndiwa, Arjen Y. Hoekstra

**Affiliations:** a International Livestock Research Institute (ILRI), P.O. Box 30709, 00100 Nairobi, Kenya; b Twente Water Centre, University of Twente, P.O. Box 217, 7522AE Enschede, the Netherlands; c International Fund for Agricultural Development (IFAD), 1191 Nile Corniche, Boulaq, Cairo, Egypt; d University of Hohenheim, Institute for Crop Science, Biostatistics Unit, 70599 Stuttgart, Germany; e Downforce Technologies, Enschede, Overijssel, Netherlands

**Keywords:** Livestock, Production, Water productivity, Land productivity, Resource use efficiency, Scenarios, Kenya

## Abstract

Population growth and rising affluence increase the demand for agricultural commodities. Associated growth in production increases dependency on natural resources in countries that attempt to meet part or all of the new demand locally. This study assesses the impact of changing meat and milk production on natural resource use in Kenya under three plausible scenarios of socio-economic development, namely Business-As-Usual (BAU), Sustainable Development (SDP) and Kenya Vision 2030 (V2030) scenarios. The IMPACT model is used to estimate projected cattle, sheep, goats and camel production parameters for meat and milk. The BAU and SDP represent standard scenarios for Kenya of a global economic model, IMPACT, while V2030 incorporates in the model features specific to Kenya's medium-term national development plan. We use calculations of water footprint and land footprint as resource use indicators to quantify the anticipated appropriation of water and land resources for meat and milk production and trade by 2040. Though camel dairy production numbers increase the most by quadrupling between 2005 and 2040, it is cattle dairy production that significantly determined gains in production between the scenarios. Productivity gains under the SDP scenario does not match the investments made thereby leading to only slightly better values for water and land productivity than those achieved under the BAU scenario. Relative to the BAU scenario, improvement in land productivity under the V2030 scenario is the most dramatic for shoat milk production in the arid and semi-arid systems but the least marked for cattle milk production in the humid system. By quantifying water and land productivity across heterogenous production systems, our findings can aid decision-makers in Kenya and other developing countries to understand the implications of strategies aimed at increasing domestic agricultural and livestock production on water and land resources both locally and through trade with other countries.

## 1 Introduction

Human population has increased globally from an estimated 2.5 billion people in 1950 to 7.8 billion people by 2020 (UN, 2019). Population growth has been most rapid in the developing world where the growth rate is spurred by high fertility rates (Spence et al., 2009). Growths in population and income as well as in urbanization have been associated more with increased demand for livestock-derived food products, than with most other food groups; and with increased livestock production and population (Alexandratos and Bruinsma, 2012; Latino et al., 2020). This so-called demand-driven livestock revolution, initially described in the early 2000s (Delgado, 2003), has been realized mostly in developing regions, especially in Asia (Thornton, 2010). The projected increase in the production of animal sourced foods, can be expected to be associated with corresponding substantial increases in land and water resource footprints and therefore to exert profound impacts on the socio-economic, political and natural resource spheres (Godfray et al., 2010; Gil et al., 2018). Consequently, sustained growth in livestock production to satisfy the increasing demand for animal source foods is contingent upon the continued availability of adequate water and land resources to support agricultural production (Wirsenius et al., 2010; Stroosnijder et al., 2012). This constraint is stronger for animal source foods such as milk and meat that require abundant freshwater for their production than for crop-based foods (Rockström et al., 2009; Mekonnen and Hoekstra, 2012).

In sub-Saharan Africa, agriculture remains the pillar of many economies and the main driver of economic growth (De Fraiture et al., 2010). Consequently, there have been repeated calls to develop and intensify agricultural production, particularly the livestock sector as envisioned in initiatives such as the Comprehensive Africa Agriculture Development Programme - CAADP (Kimenyi et al., 2013). However, agricultural intensification can adversely affect the environment, as exemplified by the livestock sector (Steinfeld et al., 2006), most particularly intensified dairy and pig production in developed regions (van der Zijpp, 1999; Gerber et al., 2005). The negative consequences of agricultural intensification have revealed a disconnect between policies that promote livestock production and the effective management of natural resources (Otte et al., 2012). This has spurred the need for accurate indicators for monitoring and evaluating the environmental footprints of food consumption (Hoekstra and Wiedmann, 2014; Bruckner et al., 2015). Water and land footprints are two such indicators used to assess the impacts of human appropriation of freshwater and land resources (Wirsenius et al., 2010; Hoekstra and Mekonnen, 2012; Liu et al., 2021).

In Kenya, the national vision for development, Kenya Vision 2030, is anchored on three pillars (economic, social and political) which the national government has adopted to achieve middle-income status by 2030 (GOK, 2007). The economic pillar of Vision 2030 aims at an annual growth rate in the gross domestic product (GDP) of 10% from 2012 to 2030. Agriculture has been identified as a key growth sector to achieving this and other goals of the economic pillar. Six strategies are identified in the Vision 2030 agricultural sector improvement plan as essential to meeting the economic pillar targets. These are: (i) Transforming key institutions in agriculture, livestock, forestry and wildlife to promote agricultural growth; (ii) increasing productivity of crops, livestock and tree cover; (iii) introducing land-use policies for better use of high- and medium-potential lands; (iv) developing more irrigable areas in arid and semi-arid lands for both crops and livestock; (v) improving market access for smallholder farmers through better supply chain management; and (vi) adding value to farm, livestock and forestry products before they reach local, regional and international markets. The social pillar seeks to establish a just and equitable society living under a secure and clean environment. The political pillar aims at entrenching and nurturing a democratic system that respects the rule of law and protects the freedom of every Kenyan. Increased prosperity in Kenya as envisioned by Vision 2030 is expected to be accompanied by increased consumption, especially of animal source foods. This will aggravate the pressure on the water and land resources required to meet the growing demand (GOK, 2007; 2010a).

The largest producers and consumers of meat and milk are located in the developed countries which is an indication of the impact of affluence on the consumption of animal source foods. For instance, beef, mutton and chevron consumption is estimated at 52 kg (kg) per person per year in the highest consuming countries (USA, Australia and Brazil), about 3 times the estimated consumption in Kenya, at 13 kg per person per year. Inclusion of poultry and pork in the estimate of meat consumption reveals even larger differences. Australia with an individual consumption of 120 kg per year still leads global consumption of all types of meat. Other countries with high consumption and production levels are USA at 116 kg per year followed by Brazil and Canada each at 92 kg per year (FAO, 2016). Consumption patterns of both meat and milk in Kenya are driven by the price, type of meat and level of processing (Ouma et al., 2000; Gamba, 2005; Bosire et al., 2017). Beef is the most commonly consumed meat product across all income groups in Kenya, due to its lower pricing compared with mutton and chevron, which are mainly consumed by the middle and high-income groups (Gamba, 2005; Juma et al., 2010). To support the higher beef consumption, around 22% of meat from cattle is imported from countries around Kenya such as Ethiopia, Tanzania and Uganda (Aklilu et al., 2002). Most production of meat for consumption is carried out under extensive production in the arid and semi-arid production systems. Milk is also produced in these areas though commercial milk production and the largest proportion of domestic milk production originates from smallholder dairy producers in the humid highlands (Behnke and Muthami, 2011; Bosire et al., 2015).

Only a few studies, mainly focused on global assessments, have thus far assessed the global environmental implications of animal source foods using land and water footprint indicators (Chapagain and Hoekstra, 2003; De Vries and De Boer, 2010; Mekonnen and Hoekstra, 2012). National analyses of the impacts of livestock production practises on the use of water and land resources are however attracting increasing attention (Ridoutt et al., 2011; Bosire et al., 2016; Dougherty et al., 2019; Mekonnen et al., 2019). The country-scale analyses enable targeted and more accurate assessments of livestock impacts on natural resource use. Due to the heterogeneity of production of animal source foods, there is a growing need for even more granular analyses targeting such spatial scales as production systems within countries. The results can then be aggregated to provide more accurate and nuanced conclusions for policy.

In this paper, we aim to assess how the livestock production policies in Kenya's Vision 2030, likely affect land and water resources in Kenya across three dominant production systems. We do this by quantifying and comparatively evaluating the environmental resources required to achieve meat and milk production projections for Kenya up to the year 2040 under three plausible scenarios.

## 2 Methods and data

To compare the Kenya government's objectives for livestock development objectives under the Vision 2030 strategy to the status-quo and, to what global objectives for sustainable development e.g., Riahi et al. (2017), indicate for Kenya and projected growth to 2040, we formulated three socio-economic development scenarios for Kenya spanning 2005–2040: the Business-As-Usual (BAU) scenario, which assumes the continuation of current policies and socio-economic conditions into the future, the SDP scenario which assumes sustainable development projections of high economic growth and low population growth, and the V2030 scenario, which assumes the full implementation of Kenya's Vision 2030 strategy (Table A.1). The year 2040 is selected as it is deemed appropriate for assessing the extent to which the strategies under Vision 2030 will have been implemented and what the outcomes will likely be. The SDP scenario is selected as there are already formulated trajectories of change in livestock production for Kenya within the model used for analysis. The scenarios differ in terms of the assumed changes in human population and income, livestock population, total meat and milk production, water and land use efficiency, feed use, and feed conversion efficiency of animals (as detailed below). We focus the assessment of the water and land footprints of meat and milk production on ruminants (cattle, sheep, goats) and include milk production by camels. Meat from pigs and chicken are excluded as the available data are insufficient to reliably calculate their environmental impacts. Camel meat is also excluded because it is economically of less importance nationally and does not feature in the framework of the global economic model used in the analysis. The scenarios for future socioeconomic development were simulated using the International Model for Policy Analysis of Agricultural Commodities and Trade (IMPACT) version 3 (Robinson et al., 2015). IMPACT provided the estimates of livestock populations, meat and milk production and consumption as well as projected prices associated with the three different future scenarios. The livestock production estimates formed the basis of our calculations of water and land footprints. Extensions to IMPACT to better account for global livestock production systems and feed types dominant in developing countries have been proposed previously but not implemented using data (Msangi et al., 2014). Our analysis fills this well identified and important knowledge gap.

### 2.1 Description of scenarios

The three scenarios were selected because they depict plausible outcomes of the policies currently pursued by Kenya and could all be integrated into the IMPACT model. The BAU scenario was already built into the model and the additional data required to operationalise the SDP and V2030 scenarios were achievable with the IMPACT model.

The BAU scenario assumes the continuation of the current trends in population and production growth up to 2040, full implementation of the currently existing legislations and no fundamental deviation from the current policies between 2005 and 2040. Population size is estimated based on decadal national censuses and projections based on fertility rates for the year 2009 (GOK, 2010b, 2012). This scenario uses projection assuming moderate economic growth.

The SDP Scenario assumes the sustainable development projection of high economic growth. Population growth is assumed to be slightly lower than under the BAU scenario. The lowered population growth rate is associated with a slightly lower effective contraceptive use rate than that under the V2030 scenario. Production increases are aimed at ensuring sustainability by enhancing resource and energy use efficiencies, improvements in production infrastructure and provision of decent wages and access to green jobs for all producers and the livestock value chain actors.

The V2030 scenario assumes full realization of the strategic goals of Vision 2030. While the Kenya Vision 2030 strategy addresses manifold issues, we focus only on human population growth, and meat and milk production, as these are the factors that we consider as having the most marked effects on livestock-related water and land use. The population growth rate is based on halving the fertility rate between 2009 and 2030 (GOK, 2012). The scenario is further characterised by a high economic growth rate and concomitant growth in agricultural and livestock production. The projected growth in production assumes increased public investment in agriculture of up to 10% of the national budget and an average annual growth rate of at least 6% in agricultural production, as outlined in the CAADP agreement (Kimenyi et al., 2013). CAADP assumes this level of growth across Africa is sufficient to eliminate hunger and reduce poverty through agriculture. The scenario further assumes the adoption of technologies and management practices that improve production. Therefore, the production growth rates assume increased budgetary allocation to agriculture. This assumption is motivated by the second and fourth strategies in the agricultural sector improvement plan in Kenya's Vision 2030. The second strategy aims to increase crop and livestock productivity whereas the fourth strategy focuses on preparing new lands for cultivation by strategically developing irrigable areas of arid and semi-arid lands for both crops and intensified livestock production.

### 2.2 The IMPACT model

The IMPACT model is designed to examine alternative futures for global food supply, demand, trade, prices, and security. IMPACT covers 62 commodities, which account for the bulk of agricultural commodities traded in world markets, including meats, milk, and eggs. A full description of the model is in Robinson et al. (2015). Aspects of the model structure relevant to this study are included in Table A1. IMPACT has been applied to analyse baseline and alternative projections of agricultural commodity supply, demand, trade, prices and malnutrition outcomes as well as to quickly evolving topics such as bioenergy, climate change, changing diet/food preferences, and many other themes (Delgado, 2003; Enahoro et al., 2018). The model's results on ruminant animal numbers (cattle, sheep and goat) and the (total) production or yields (per animal) of meat and milk as well as model projections of feed production and use from crop sources are relevant for understanding contemporary and projected future trajectories of meat and milk production. Extensions to IMPACT to better account for global livestock production systems and feed types used to represent these systems in developing countries have been proposed previously but not implemented using data (Msangi et al., 2014). Our analysis thus fills this well identified and important knowledge gap. Input data for IMPACT for the two standard scenarios, i.e. BAU and SDP, are available on GitHub at https://github.com/IFPRI/IMPACT. For the Kenya vision scenario, indicators of the key drivers of economic change (mainly human population and income) were replaced for the status-quo scenario, with parameters representative of the Kenya Vision 2030 strategy (Appendix, Table A.1).

Water use in crop production is accounted for in the IMPACT model but not water use in other feed production, or direct use by livestock. The impacts of livestock production on land resources are also not accounted for. We thus use relevant results from the model to calculate water and land footprints of future livestock production in Kenya that are associated with open international trade in agricultural and livestock commodities. We simulate three socio-economic development scenarios and estimate water and land productivities of ruminant meat and milk production for each.

### 2.3 Feed amount and their composition in the systems

We disaggregated the national estimates of feed demand generated from the IMPACT model into the underlying agricultural or livestock systems contributing to livestock production (Bosire et al., 2015); while accounting for feed types other than crops and feed grain concentrates (ILRI, 2010).

Feed consumption patterns vary in Kenya's production systems (ILRI, 2010). We estimate the feed use in the three production systems by determining the amount used annually or till slaughter based on diet composition and quality, feed conversion efficiency and milk, or meat production or both per animal. The total feed consumed by each animal category in each system is estimated by combining information on feed composition, feed conversion ratio and product yield within each production system estimates of livestock numbers derived using IMPACT.

Feed volumes consumed per animal for both meat and milk production in each production system were estimated following Mekonnen and Hoekstra (2012):

(1) $$Feed\left[a,s,p\right]=FCR_{a,s}\times P_{a,s}$$

Feed[a,s,p] (ton/yr) is the total annual or till-slaughter feed consumed by an animal in category a in production system s*,* $FCR_{a,s}$ is the feed conversion ratio (kg dry mass of feed/kg product) for an animal in production system a and $P_{a,s}$ (kg/yr) or (kg) is the amount of product (milk, meat) produced by animal a in production system s. The feed conversion ratios were taken from Bosire et al. (2019) and represent improvements in animal management within the V2030 and SDP scenarios in terms of breeds, feeding and range management as envisioned in these scenarios.

Feed is categorised into four classes: (i) pasture, which includes hay and silage; (ii) planted forage; (iii) crop residues; and (iv) compounded feed and supplements (ILRI, 2010). The proportion of each feed type in each of the production systems was also derived from the FEAST tool for representative study sites (ILRI, 2010).

### 2.4 Estimation of animal numbers and milk and meat production in each system

We derived numbers of cattle, sheep and goats for each of the three production systems and scenarios using the IMPACT model, and camel numbers from published studies (Behnke and Muthami, 2011). IMPACT was used to derive estimates of country-level numbers of slaughtered (cattle, sheep and goats) and milked (cattle) animals for Kenya that are consistent with global market (price and supply) conditions (Robinson et al., 2015). System-level disaggregation of the national estimates into (three) livestock production systems, and the accounting for camel production relied on information from the literature (Behnke and Muthami, 2011; Bosire et al., 2015). The animal numbers were used to quantify the expected total meat and milk production, feed intake, land and water footprints and the water and land productivities for each system. Camel numbers are not modelled in IMPACT and were not used in estimating meat production for either the production systems or scenarios because camels’ contribution to the total meat production in Kenya is still relatively low (Behnke and Muthami, 2011; Bosire et al., 2015).

### 2.5 Assessment of water and land productivity of livestock production

The water and land productivity of livestock production in the three scenarios are estimated following Mekonnen et al. (2019):

(2) $$WP_{prod}\left[a,s,\;p\right]=\frac{PO}{WF}\;\left[a,s,p\right]$$

where $WP_{prod}\left[a,s,\;p\right]$ is the water productivity of product p*,* from animal a in each system of livestock production s, PO[a,s,p] is the production of product p per animal in each production system (kg/animal) and is generated as a model output for each scenario using IMPACT, *WF* [a,s,p] is the water footprint of the animal per year for milk production or until slaughter for meat production (m^3^/animal). The water footprint is an indicator of direct and indirect appropriation of freshwater resources used in production (Hoekstra et al., 2011). We consider both the blue water footprint, indexing the consumption of blue water resources (groundwater and surface water), and green water footprint, representing the consumption of green water resources (rainwater in the soil).

Land productivity $LP_{prod}\left[a,s,p\right]$ in the three production systems is also estimated using similar assumptions as the water productivity as follows:

(3) $$LP_{prod}\left[a,s,p\right]=\frac{PO}{LF}\;\left[a,s,p\right]$$

where *PO* [a,s,p] is the production of product p per animal in each production system (kg/animal), LF[a,s,p] is the land footprint of the animal per year for milk production and until slaughter for meat production (ha/animal). The land footprint is defined here as the actual land used from either production or consumption point of view (Erb, 2004). We distinguish between two components in the land footprint: grazing land and cropland.

#### 2.5.1 Assessment of water and land footprints of feed

It follows that the water footprint of an animal can be expressed in terms of m^3^/yr/animal, or, when summed over the lifetime of the animal, in terms of m^3^/animal. The water footprint of an animal can thus be expressed as:

(4) $$WF\left[a,s\right]=WF_{feed}\left[a,s\right]+\;WF_{drink}\left[a,s\right]+WF_{service}\left[a,\;s\right]$$

where, the water footprint of an animal in category *a* in production system *s,* WF[a,s], sums the footprints related to consumption of feed $\;WF_{feed}\left[a,s\right]$, drinking water, $WF_{drink}\left[a,s\right]$ and service water, $WF_{service}\left[a,s\right]$. The water footprint of feeds contributes the most to the total water footprint of the animal (Mekonnen and Hoekstra, 2010).

The land footprint of each animal is expressed as the land required to produce the feed consumed by each animal in each system in ha/animal. This can be expressed as:

(5) $$LF\left[a,s\right]=LF_{feed}\left[a,s\right]$$

where LF[a,s] is the land footprint of an animal in category *a* in production system *s,* related to feed production. We do not consider the land used for housing the animals or feed storage as this is difficult to estimate at the scale of this study and is not consistent across farms.

The water footprint of feed demand is estimated as follows:

(6) $$WF_{feed}\left[a,s\right]=\overset{n}{\underset{p=1}{\sum }}\left(Feed\;\left[p\right]\times wf_{feed}\left[p\right]\right)$$

where Feed[p] is the total feed of type p that each animal category a consumes (tonne/animal), $wf_{feed}\left[p\right]$ is the water footprint of feed crop of type p (m^3^/tonne) in each system. The water footprint of the feed crops in each of the production systems was derived from estimates in Bosire et al. (2019).

The land footprint of feed was similarly estimated for each animal in each of the production systems for the three scenarios as follows:

(7) $$LF_{feed}\left[a,s\right]=\overset{n}{\underset{p=1}{\sum }}\left(\frac{Feed\;\left[p\right]}{Y_{a,s,p}}\right)$$

where Feed[p] is the total amount of feed of type p that each animal of category a consumes (tonne/animal), $Y_{a,s,p}\;$ is the yield of the feed crop or crop of type p (tonne/ha) in each system.

## 3 Results

### 3.1 Livestock population changes during the period 2005–2040

**Figure 1:**
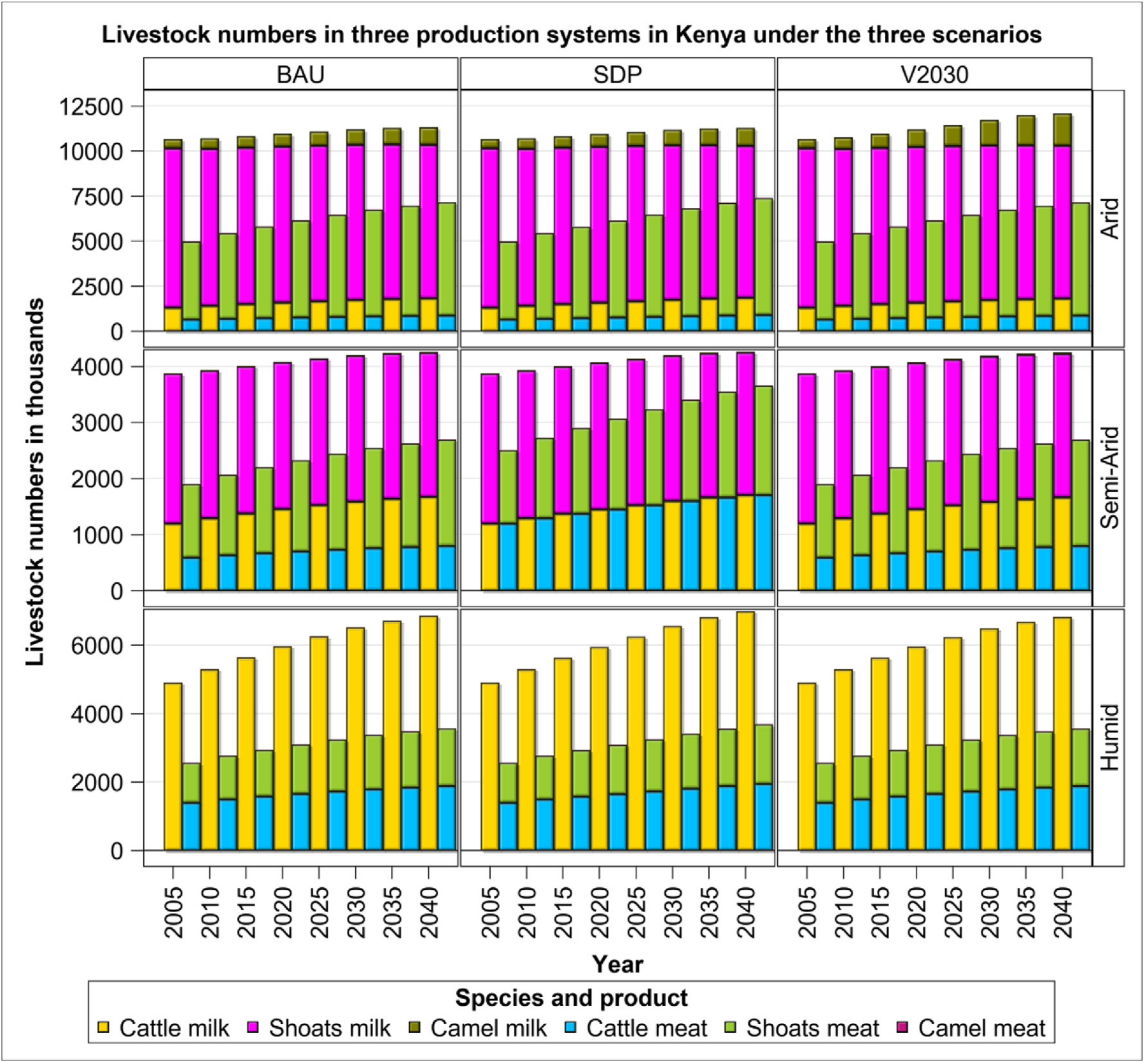
Numbers of cattle, sheep and goats (shoats) and camels in the three Kenya production systems and scenarios during the period 2005–2040.

The numbers of cattle, sheep, goats and camel increased from 2005 to 2040 under all the three scenarios by 15–19% for the dairy herds and 42–47% for the meat producing animals (Figure 1). However, the absolute and percentage increase in numbers of cattle, sheep, goats and camel between 2005 and 2040 differ between both the production systems and scenarios. Regarding milk production, the percentage increase was the greatest under the V2030 scenario (19%) and the least under the BAU scenario (12%). The absolute numbers of animals raised for meat production similarly increased across all the three scenarios but the most in the SDP scenario (47%) and at lower but comparable rates (42%) for both the BAU and V2030 scenarios (Figure 1).

For milk production, camel numbers increased the most, more than quadrupling during 2005–2040 in the V2030 scenario. However, dairy camels almost doubled in both the BAU and SDP scenarios. The increase in dairy cattle herd is mainly associated with lack of improvements in productivity. For meat production, sheep and goats increased the most (50%) from 2005 to 2040 in the SDP scenario but by a somewhat lower amount (45%) in both the BAU and V2030 scenarios. Though the cattle reared for meat production is a third that of sheep and goats, it also increased discernibly in the SDP (40%), BAU (35%) and V2030 (35%) scenarios. Cattle also increased (39–42%) but at much lower rates than camels (95–256%) from 2005-2040, with the highest growth for cattle recorded for the SDP scenario. Sheep and goats showed the opposite trend with a slight decline in the dairy herd by 2040, a pattern consistent across all the scenarios and reflective of improvements in productivity of dairy sheep and goats (Figure 1).

Among the three systems, absolute livestock numbers were the lowest for the arid system, followed closely by the semi-arid system, and the highest for the humid system regardless of scenario. Cattle numbers are the highest in the humid system, intermediate in the arid system and the least in the semi-arid system across all the scenarios. Dairy sheep and goats dominate both the arid and semi-arid systems, contributing 2–7 times as many animals as cattle or camels. There are no dairy sheep and goats or camels in the humid system. Though dairy camels increase over time, by as much as 72% in the V2030 scenario, their absolute numbers are too few to make a substantial contribution to the overall milk production in Kenya. The dairy livestock numbers grew little despite the relatively large increase in overall livestock numbers. Shoat numbers greatly affect the overall number of both the meat and milk producing livestock as even a small decrease in their numbers leads to a lowered overall growth in production in each system. In conclusion, dairy cattle, sheep and goats increased strikingly between 2005 and 2040 in all the three scenarios but did not increase substantially faster in either the SDP or V2030 scenario relative to the baseline scenario in all the three production systems.

### 3.2 Production of meat and milk between 2005 and 2040

**Figure 2:**
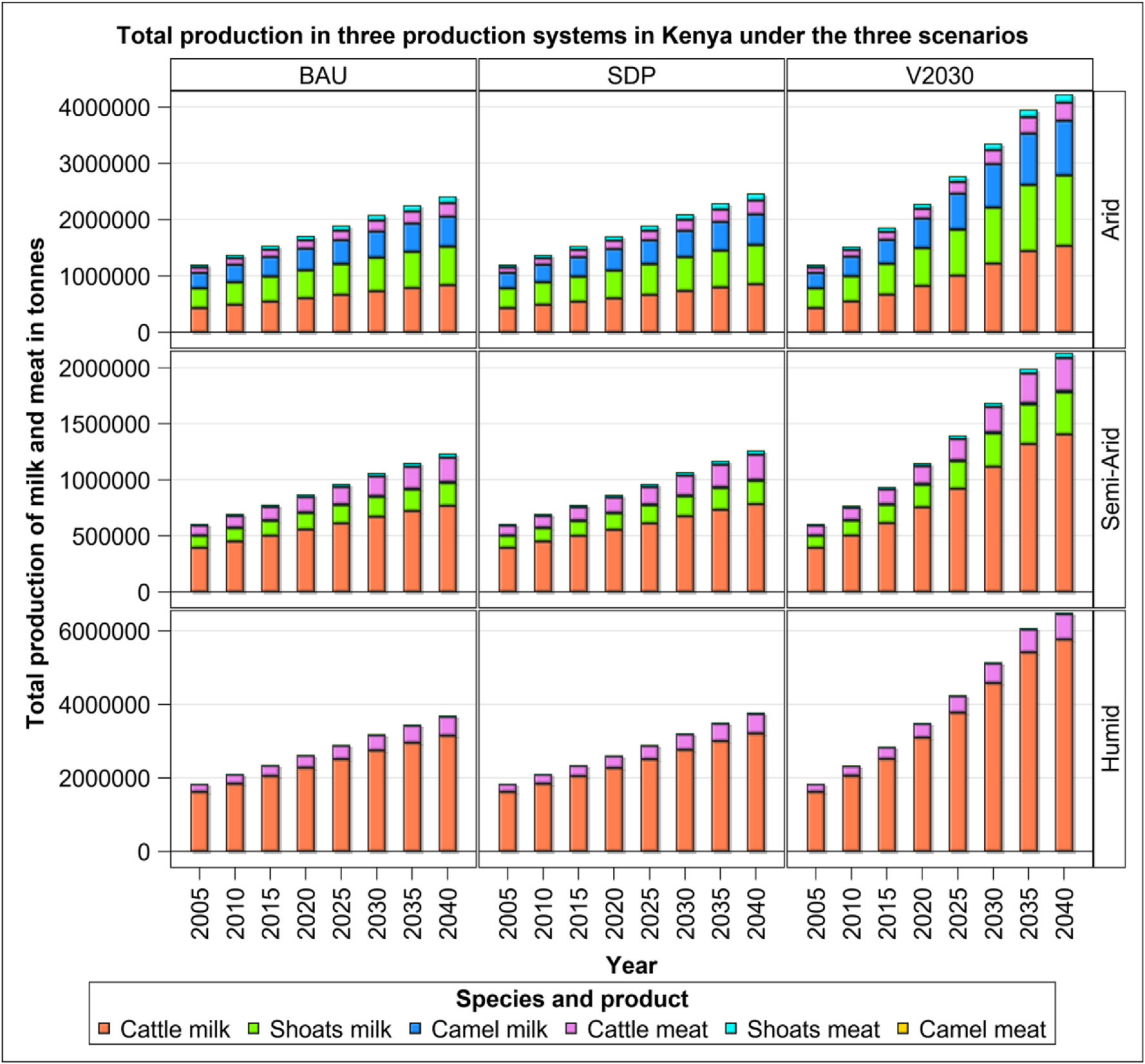
Production total for all the scenarios, commodities and timeframes.

Milk and meat production increases across all the three scenarios but at rates that differ across scenarios, with the V2030 scenario displaying the fastest growth (Figure 2). Milk production increases the most from 2005 to 2040, more than quadruples under the V2030 scenario and doubles under both the BAU and SDP scenarios. Meat production shows a similar trend and quadruples under the V2030 scenario but grows by a larger margin than milk production, more than doubling under both the SDP and BAU scenarios. The growth in total milk production between 2005 and 2040 is only slightly higher under the SDP than the BAU (2%) scenario but is strikingly higher under the V2030 scenario than under either the BAU (83%) or the SDP (80%) scenario. Likewise, the difference in growth in meat production is less pronounced between the scenarios and is only marginally higher under the SDP relative to the BAU (3%) scenario and mildly higher under the V2030 scenario than either the BAU (24–35%) or the SDP (20–35%) scenarios.

Among the species, cattle dominate milk production in all the production systems and scenarios. In the arid system, cattle produce more milk than either sheep and goats (19%) or camels (37%). In the semi-arid system cattle similarly produce significantly more milk than either sheep and goats (73%) or camels (400%). Cattle also produce significantly more meat than sheep and goats across all the production systems and scenarios. Across systems, the excess meat produced by cattle relative to sheep and goats increases dramatically with humidity and is the lowest for the arid (50%), intermediate for the semi-arid (83%) and the highest for the humid (94%) systems.

### 3.3 Water and land footprints of milk and meat production in Kenya during 2005–2040

**Figure 3:**
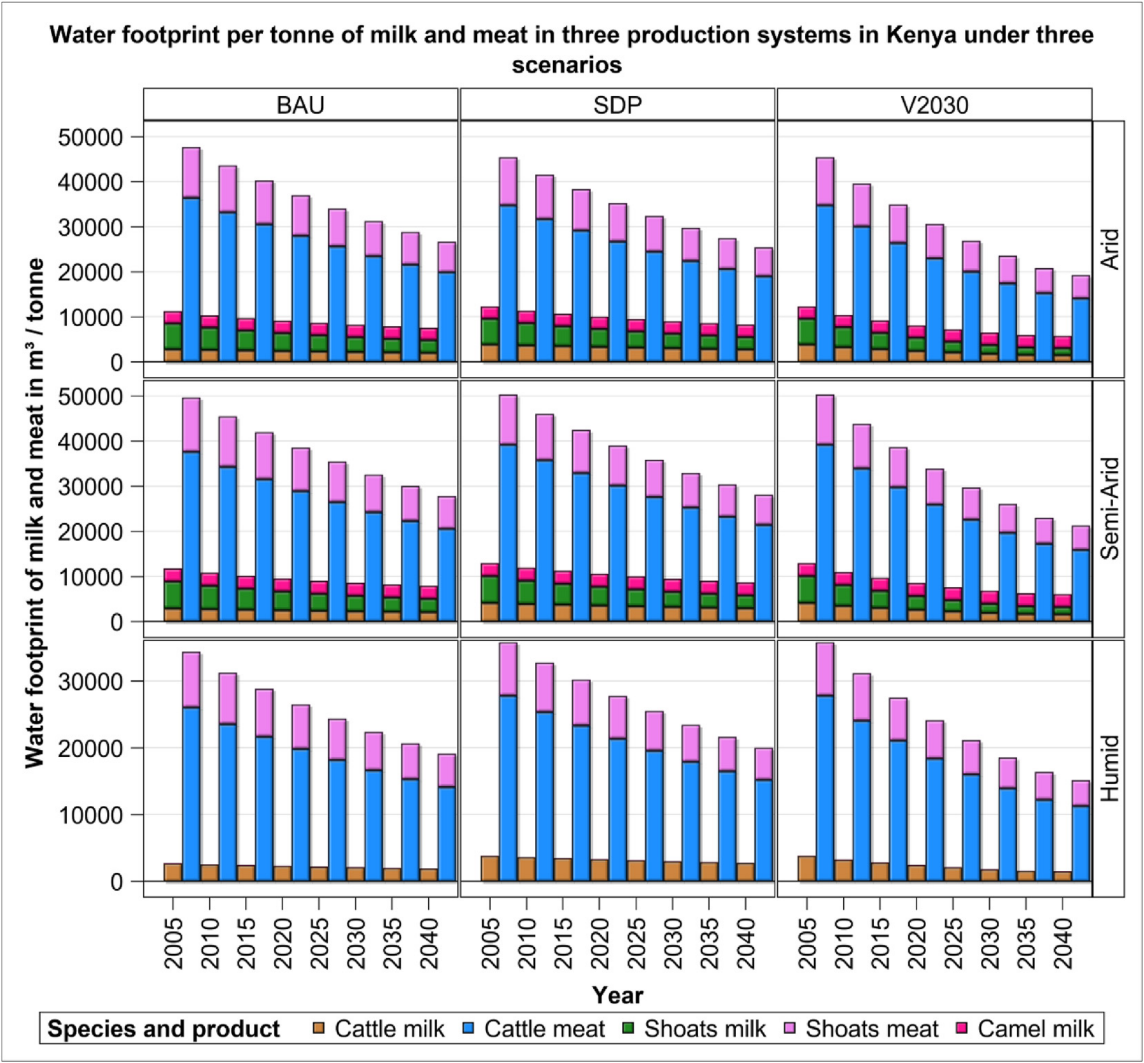
Water footprint of cattle, sheep and goats (shoats) and camels in three production systems and scenarios during the period 2005–2040.

The water and land footprints of milk and meat per tonne decreases persistently and markedly from 2005 to 2040 regardless of system or scenario, indicating increasing water and land use efficiency. However, the efficiency gains vary with the system, scenario, and livestock species (Figure 3). The water footprint per tonne of milk and meat decreases over time. The rate of decline in water footprint is the fastest for the V2030 scenario, resulting in the lowest water use in all the systems by 2040. In the humid system one tonne of milk will require 22% less water to produce in 2040 under the V2030 than under the BAU scenario. Cattle achieve a substantial gain in water use efficiency in milk production in all the three systems under the V2030 (46%) than under the SDP scenario. Sheep and goats similarly gain 44% in water use efficiency of milk production under the V2030 than under the SDP scenario in the semi-arid system. This is mainly attributed to improvement in milk production per sheep and goats (shoats) under the V2030 scenario. The change in water footprint between the BAU and SDP scenarios is less marked because of little improvement in milk production under both scenarios. The water footprint of camels changes little because there are no interventions for improving camel milk production under any of the three scenarios.

Overall, the water footprint of meat production is lower for sheep and goats than cattle in all the systems. The water footprint per tonne of meat is the lowest for sheep and goats in the humid system under the management practices incorporated in the V2030 scenario. Producing meat in the semi-arid systems, though expected to be more water use efficient than in the arid systems due to more evaporative water loss in arid systems, has a larger (7%) water footprint than in the arid system across all the scenarios. This indicates lower efficiency in the semi-arid system than in the arid system.

**Figure 4:**
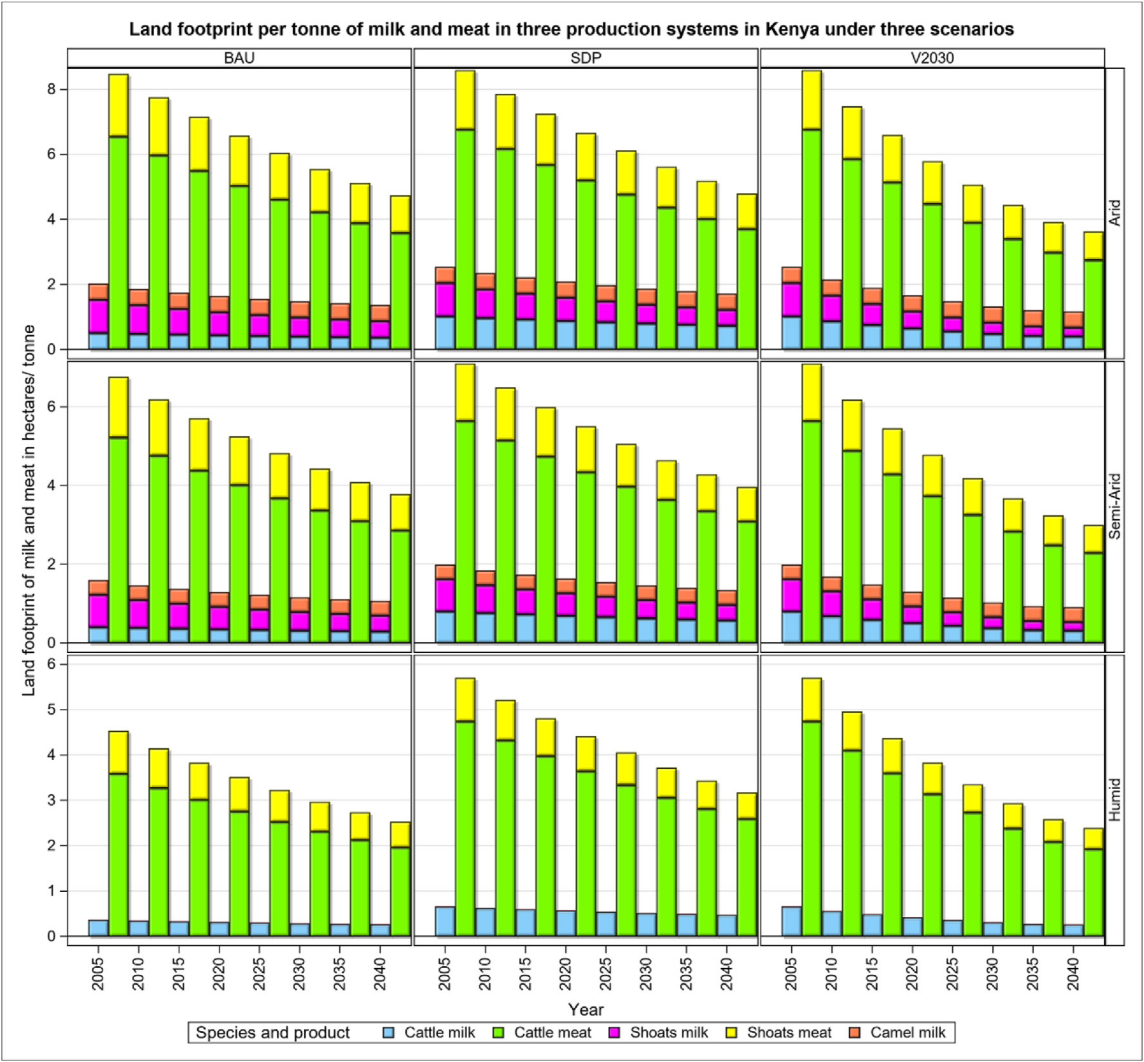
Land footprint of cattle, sheep and goats (shoats) and camels in three production systems and scenarios during the period 2005–2040.

The land footprint of milk production shows trends similar to those for the water footprint, with the largest land footprint estimated for the SDP scenario (Figure 4). This is mainly due to lower animal productivity that does not match the better management practices built into the SDP scenario relative to the BAU scenario. But higher productivity under the V2030 scenario leads to a better land footprint (23%) than under the SDP or BAU scenarios. The inclusion of more feed crops in the diets in the SDP scenario leads to a higher cropland footprint for similar amounts of forage and compounded feeds in the diets of cattle under the SDP than the V2030 scenario.

### 3.4 Changes in water and land productivity across production systems in Kenya during 2005–2040

**Figure 5:**
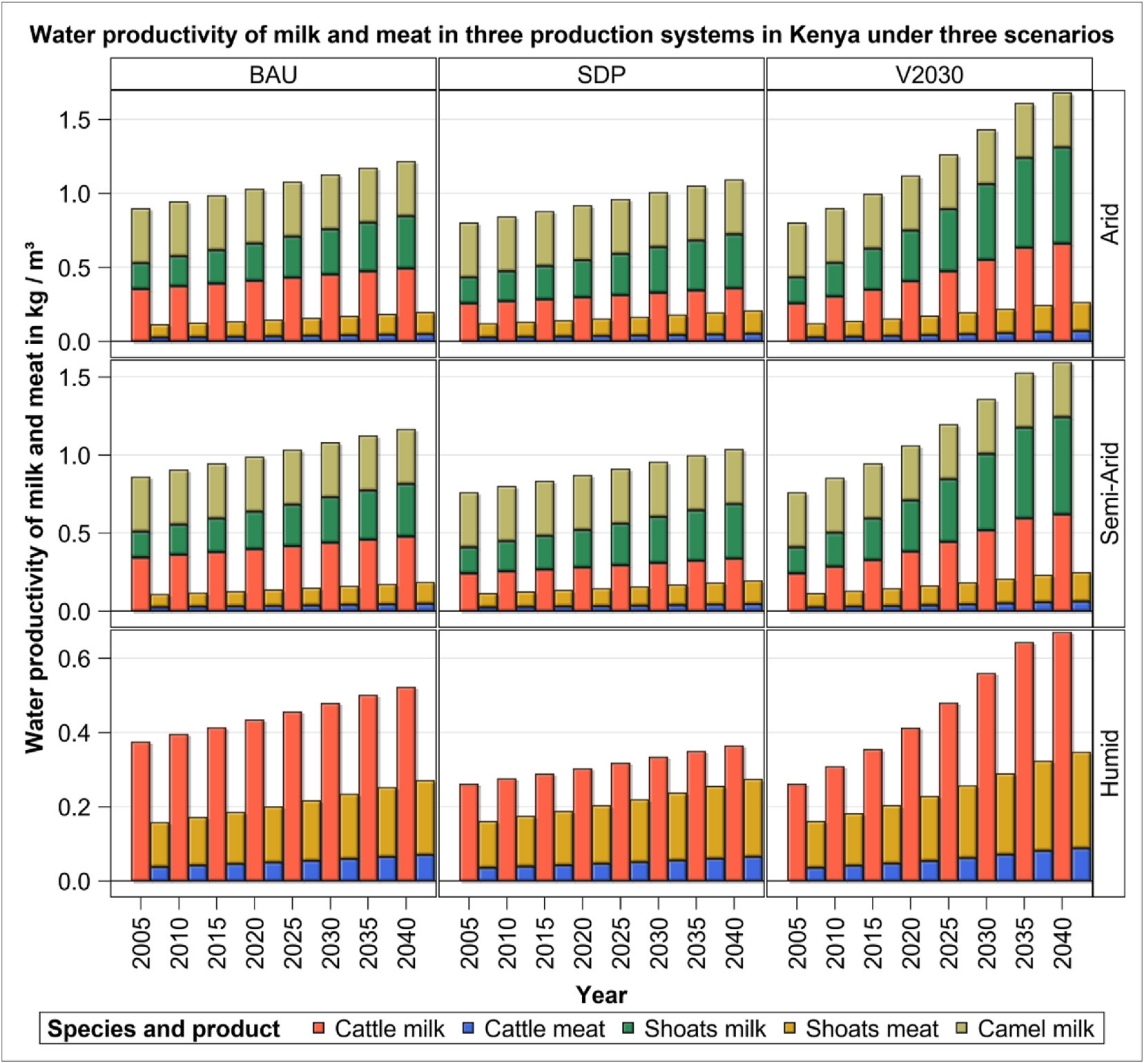
Water productivity of cattle, sheep and goats (shoats) and camels in three production systems and scenarios during the period 2005–2040.

**Figure 6:**
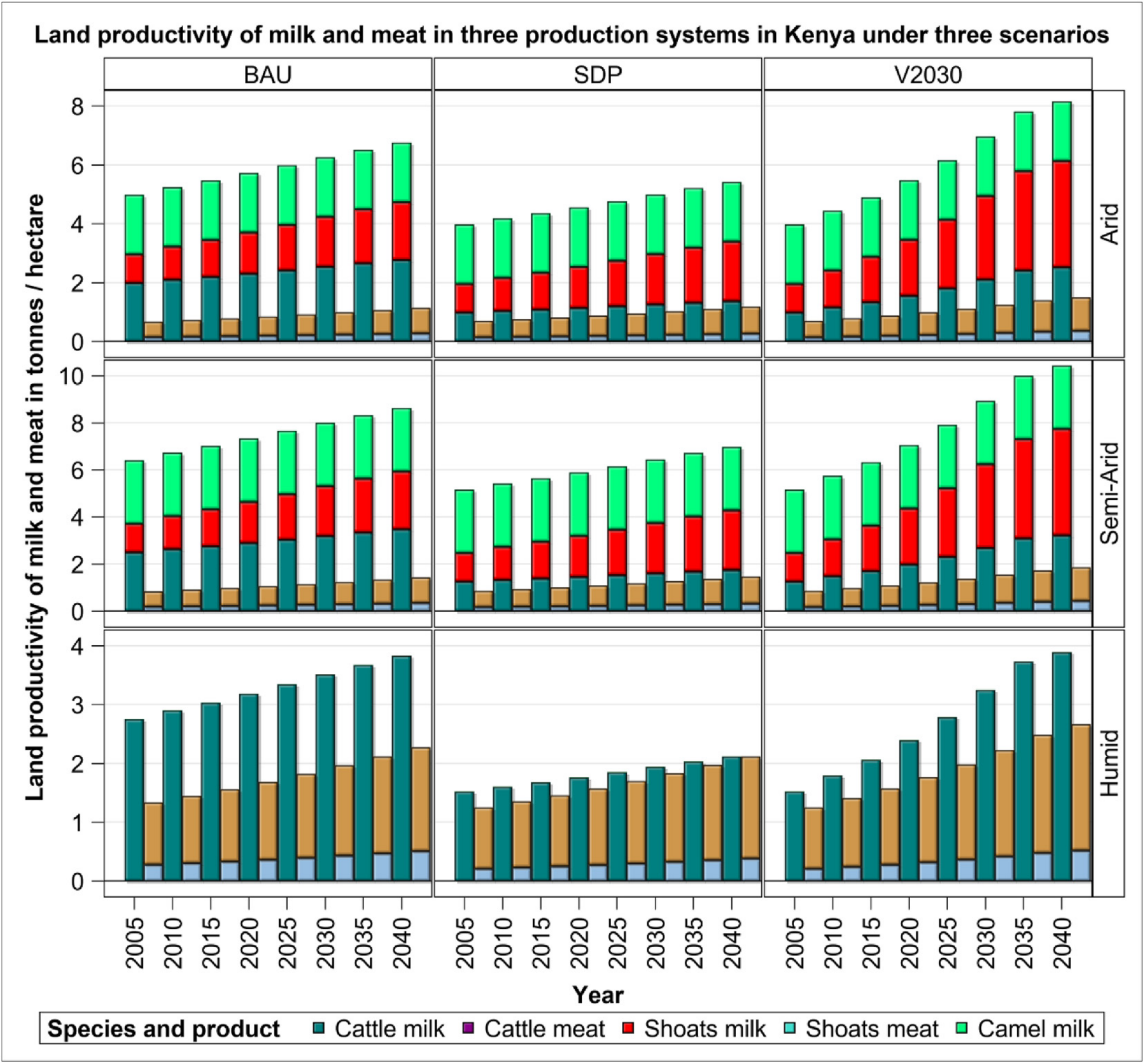
Land productivity of cattle, sheep and goats (shoats) and camel in three production systems and scenarios during the period 2005–2040.

Water and land productivities of meat and milk increase persistently and strikingly from 2005 to 2040 for all livestock species across all systems and scenarios (Figures 5 and 6). The water productivity of meat and milk ranges from 0.027 kg/m^3^ for beef to 0.6 kg/m^3^ for milk production by sheep and goats between 2005 to 2040 (Figure 5). Overall, water productivity improves the most between 2005 and 2040 under the V2030 scenario (by up to 84%) and is more pronounced for milk than meat production. Water productivity for cattle in 2040 is the largest under the V2030 and the least under the SDP scenario. Across all the scenarios, production systems and species, water productivity of milk is the highest under the V2030 scenario (by up to 78–85%) relative to the SDP scenario. For cattle, the water productivity of milk production is only mildly higher under the V2030 than the BAU (28%) scenario in the humid system. The water productivity of meat production is similarly the highest under the V2030 scenario compared with the BAU and SDP scenarios. Meat production by cattle and sheep and goats also exhibits small differences (5–7%) between the BAU and SDP scenarios for all the three systems.

Among the livestock species, water productivity of cattle milk is consistently higher than that of sheep and goats or camels. However, the water productivity of milk production in the humid system by sheep and goats increases almost four-fold to about 0.6 kg/m^3^ from 2005 to 2040 under the V2030 scenario similar to that by cows. Across all systems and scenarios, the water productivity of meat production by sheep and goats is 3–4 times that for cattle.

For land productivity, the amount of milk and meat produced per hectare of land, improves between 2005 and 2040 but at rates that vary noticeably across systems, scenarios and livestock species (Figure 6). The improvement in land productivity is the most dramatic for shoat milk production in the arid and semi-arid systems under the V2030 relative to the BAU (73%) scenario and is the least marked for milk production by cattle in the humid system under the V2030 relative to the BAU (2%) scenario (Figure 6). The land productivity of meat does not increase as much between the V2030 and BAU scenarios as that of milk. The largest increases in land productivity between 2005 and 2040 is for beef (35%) between the SDP and V2030 scenarios and shoat meat (29%) between the BAU and V2030 scenarios. Species-specific improvements in land productivity are similar to those for the corresponding water productivity, with the largest improvements in productivity due to the quadrupling of sheep and goat meat production between 2005 and 2040. Variations in the water and land productivities are primarily due to variations in the feed conversion ratio, the proportion of different feed types in the overall diet, and the productivity of the animals as well as the feed in the production systems.

## 4 Discussion

### 4.1 Production changes under the three scenarios

Increased demand for livestock products as projected by various studies on the developing world must be met by increased production or imports (Delgado, 2003; Herrero et al., 2017). However, the increase in production needs to be carried out in a way that does not adversely affect the environment and ensures equitable distribution of gains across all actors (Conceição et al., 2016). An increase in production often forms a critical component of efforts to alleviate poverty as shown by the agriculture-led growth in Africa, where agriculture is seen to be more than twice as effective in reducing poverty as industry-led growth. As envisioned in the Kenya's Vision 2030 agricultural development strategy and outlined by Wiggins (2016), the key to sustaining and enhancing growth in agricultural performance in Kenya will most likely lie in increasing smallholder productivity and investing in developing non-farm activities. The agricultural sector in Kenya experienced an average annual growth rate of 3.5% in the 1980–1990 which declined to a low of 1.3% in the 1990s (Ndung'u et al., 2011). This drop was mainly as a result of mismanagement, decline in investments by the government from 13% of the national budget in the 1980s to a low of 2% in the 1990s, collapse of some key agricultural institutions and, breakdown in coordination of agricultural extension and research (GOK, 2010a). However, the improvements in the agricultural sector in the 2000s is evidenced by an improved annual growth rate to 2.4% during 2000–2007. This was due to the government's increased investment in agriculture of 2%–4.5% of the national budget. A 2008 drop to -2.5% and the subsequent attempt to revitalise the sector is reflected in the rather low and slow growth in production demonstrated in the BAU scenario.

Projections under the SDP scenario are also not very impressive and only a slight increase in the productions of milk by 2% and meat by 3% above that in the BAU scenario, is observed. This indicates that the strategies for achieving improved livelihoods and wellbeing in the SDP scenario are less likely to lead to great improvements in production or meet the food security goals of Kenya. However, the much larger improvements in the production of both milk and meat under the V2030 scenario, which increase milk production by almost 200% and meat production by 20–35% relative to the BAU or the SDP scenario, presume greater investments and projected improvements in productivity as championed in the Kenya's Vision 2030 agricultural development strategy.

These improvements in the livestock sector are contingent upon the production of sufficient quantities of compounded livestock feeds. Increasing livestock productivity should therefore also entail concurrently increasing productivity in the crop sector, increasing feed crop quality, and decreasing water and land demand per unit of feed. In Kenya, maize is the major component of compounded livestock feeds, as well as the main staple food. Maize productivity is unfortunately declining in many parts of Kenya despite the rising demand, due to widespread land subdivision, land degradation through soil erosion and other factors (Jones and Thornton, 2009; Maitima et al., 2009). To meet both the food and feed demands in Kenya, it will thus be necessary for farmers to be supported to increase productivity of cereals and other noncereal feedstock like fodder to levels higher than those envisioned in the Kenya's Vision 2030 strategy and the Agricultural Sector Development Strategy. Realizing the potential for large-scale production of feed crops such as maize and wheat may lie in using the less exploited areas like Turkana and Tana River, located in the arid and semiarid production systems in Kenya, where farmers may use their excess produce to make animal feed and where land subdivision is still relatively less extreme. However, meeting the water needs for the increased feed crop production in the already water-stressed arid and semi-arid production systems would likely be exceedingly challenging in contexts of rapid human population growth and the projected temperature rise and widening rainfall variability linked to global warming (Enahoro et al., 2018; Kozicka et al., 2021). Moreover, achieving and sustaining the projected increases in feed crop production would strongly depend upon limiting policy vacillation.

### 4.2 Options for improving productivity

Additionally, a country may decide to import products that can be produced at a relatively low cost or more efficiently by another country, and therefore are sold at lower prices. In both cases, meeting the deficit through imports will require that the source areas are able to produce surpluses and that the country requiring the product is able to purchase the commodities (Bourguignon and Morrisson, 1990). It is noteworthy, that some countries are able to produce surplus dairy and meat (such as Brazil, India and New Zealand) which are, in some instances, imported into Africa (Herrero et al., 2014). Kenya imports very low quantities of milk which is mainly in powder form (EADD, 2008). However, both formal and informal meat imports in the form of live animals, were equivalent to around 40% of the total demand in the 2000s, the latest period for which data are available (Aklilu et al., 2002; Aklilu, 2008). To ensure that Kenya can purchase the shortfall in meat supply, incomes in the country will need to grow in tandem with the gaps between demand and domestic production.

Another option will be to further increase domestic livestock production beyond the V2030 scenario's projections. In Africa, currently ranked as the continent with the lowest productivity of livestock production in the world, there is still a large potential to improve productivity (Tittonell and Giller, 2013) by improving breeds, the quality of feeds, or both. Our assessments showed there would still arise a need to import meat and milk into Kenya, under both the SDP and V2030 scenarios. Productivity will thus need to improve even faster than under the ambitious high productivity V2030 scenario for domestic production to close supply gaps without exerting substantial new pressures on the water and land resources. However, several processes will likely diminish the likelihood of substantially enhancing livestock productivity. These include rapid population growth, with the Kenyan population doubling every about 20 years, that is leading to higher pressures on land and reduced fallow periods, and limited viable options open to farmers who are forced off their farms (Headey and Jayne, 2014).

### 4.3 Water and land footprints associated with the three scenarios

In Kenya, both meat and milk production come mainly from grazing land. Our results show that green water footprints dominate (Table S1), and have lower opportunity costs than cropland and blue water, especially in the arid and semi-arid regions (green water is the rainwater consumed while blue water is the volume of ground and surface water evaporated (Hoekstra et al., 2011)). However, cropland footprints and blue water footprints increase under both scenarios, an outcome related to the increasing use of irrigation for feed crops (Table S2). The increase in the proportion of compounded and supplemental feeds in livestock leads to increase in meat and milk yields (Herrero et al., 2013). Supplying this increased proportion of compounded and supplemental feeds however translates into an increased crop land footprint, with the potential to increase the competition for arable land in food crop production. In the V2030 scenario, with a relatively high proportion of feed crops in the livestock diets, the risk of this type of conflict is higher than in the BAU or SDP scenario. Concerning the water footprints of meat and milk productions, increasing the production of maize through irrigation in arid and semi-arid areas, which is also partly used as livestock feed, would increase the blue water footprint in both crop and livestock production. These production systems in Kenya are already blue water scarce (Hoekstra et al., 2012) and so increasing the use of irrigation would further elevate this scarcity and escalate the ongoing conflicts fuelled by water scarcity. An additional factor to consider is the implications of increased production on the overall water and land footprints in Kenya. The total water and land footprints of production of milk and meat, increases over time in all the scenarios, but does not differ significantly between the scenarios. This lack of difference reflects the interplay of several other factors other than improved production contributing to the improved water and land footprints. For instance, improved production may create unsustainable production hotspots (Liu et al., 2008). To mitigate against these and ensure that environmental sustainability goals are simultaneously met, meat and milk production must ensure that ecological restoration is carried out in the degraded hotpots and that future production objectives factor in ensuring sustainability (Ibidhi and Salem, 2020; Liu et al., 2020). To better contextualise the implications of improved production between the scenarios, we compare the water and land productivities in section 4.4. below.

### 4.4 Water and land productivities associated with the scenarios

Water productivity is inversely related to water footprint and is a good measure of water use efficiency across production systems and countries. An improved productivity trend is seen in the projected milk production under the V2030 scenario and an increase of only 19% in the number of milk producing animals. There is still room to increase productivity to the higher levels reported in Mekonnen et al. (2019) for cattle production. Even with improved productivity under the V2030 scenario, the values for Kenya are only comparable to those for milk water productivity in the USA in the 1980s which approximated the 0.67 kg/m^3^ achieved under the V2030 projections for 2040. To achieve the even higher productivity levels, there will be a need to invest in improving the feed further, livestock productivity through genomic breeding programs and the feed crop yields (Blümmel et al., 2003; Descheemaeker et al., 2011; Mayberry et al., 2017). Camel milk productivity is similar to that for cattle and therefore provides an alternative to meeting milk needs in the arid and semi-arid systems where they are most efficient. However, improved water productivity of beef is better in 2040 than under those assessed by Mekonnen et al. (2019) for 2020. This is especially so for the V2030 scenario where the value even reaches a high of 0.08 kg/m^3^ in 2040. This is realized in the humid system, where there has been the greatest investments in improving milk production over the last few decades in Kenya (Omore et al., 1999; Place et al., 2006). Though slightly lower, the beef production efficiency in the arid and semi-arid systems also shows great improvements, indicating that with improved investments as outlined in the V2030 scenario, it is possible to close productivity gaps in Kenya and other developing countries facing similar constraints to increased production.

Sheep and goats also demonstrate very high meat and water productivities which are about 10 times those for cattle. This, combined with their higher land productivity for both milk and meat than cattle in the arid and semi-arid systems, is the main important aspect to focus on as they are more efficient than are cattle at using the land. This supports the increased focus on production of sheep and goats for export as they have better meat productivities than cattle in the arid and semi-arid systems. This further reenforces the strategy to increase investments in the arid and semi-arid systems where most of the meat is produced. The trend towards more sheep and goats in these areas since the 1970s due to more frequent and intense droughts (Bosire et al., 2015) also supports the need for increased consideration of the role of sheep and goats and camels in the adaptation strategies in these production systems.

## 5 Conclusion

If the budget for agriculture is increased from its current level to meet the recommendations of the Comprehensive Africa Agriculture Development Programme, then meat and milk production in Kenya in 2030 can be expected to grow 1.5 to 2 times faster. However, the production increase would be insufficient to meet the projected growth in demand for these two products, diminishing the prospect of achieving the self-sufficiency hoped for in Kenya's policy strategy. However, it may be possible to achieve self-sufficiency in meat and milk production under a more modest increase in human population numbers than that forecasted by the V2030 scenario. The projected growth in livestock population and in meat and milk production under the SDP and V2030 scenarios does not match the growth in consumption, leading to a widening gap between local supply and demand for these two products.

To meet the meat demand, it is worthwhile considering increasing meat imports from Kenya's neighbouring countries or increasing production in the humid production system where the economic costs of production are lower, but competition with staple crops is greater. Because meat has lower economic water and land productivities than milk, it is worthwhile for Kenya to consider importing meat and enhancing milk production, especially in the humid systems, to meet the rising demand.

Strategies that focus on increasing livestock and crop productivities reduce water and land footprints per unit of production, creating room for increased production. However, land and water resources in Kenya are already scarce and overexploited in many regions. Besides, climate change, notably rising temperatures and widening rainfall variability, may further adversely impact the future availability of water resources.

The meat and milk productivities of water and land are better under the scenario that considers the implementation of the Kenya Agricultural Development Strategies than will obtain from current trends, or the Sustainable Development Projections scenarios. This can be explained by the much larger investment in improving the feed yields though precision irrigation and yield development, increased involvement of research and extension services, which lead to increased production per animal, and better feed conversion ratios. The Vision 2030 strategy for improving livestock production in Kenya is important for reducing the speed with which the environmental footprint of the sector will increase, by improving the water and land productivity locally.

The milk and meat production estimates are disaggregated over three production systems in Kenya. Each system has a varying range of conditions, bio-physical and socio-economic, that favour the production of either meat or milk and plausible changes suggest different trends per production system. Future research could thus focus on furthering this scale of analysis to ensure that fine-scale data are available for integration into models. This will allow for targeted projections and development of area-specific policy decisions as trends differ amongst the meat and milk production systems and should additionally allow for incorporation of climate change impacts and nature conservation on the projected patterns.

For simplicity and brevity, we did not consider the possible effects of climate change and the COVID 19 pandemic nor compare our projections of meat and milk production with the total available water and land. We do however recommend consideration of these factors in future analyses when more accurate data become available.

## Declarations

### Author contribution statement

Caroline K. Bosire; Dolapo Enahoro: Conceived and designed the experiments; Analyzed and interpreted the data; Wrote the paper.

Nadhem Mtimet; Joseph O Ogutu: Analyzed and interpreted the data; Wrote the paper.

Maarten S. Kro; Jan de Leeuw; Arjen Y. Hoekstra: Conceived and designed the experiments; Contributed reagents, materials, analysis tools or data; Wrote the paper.

Nicholas Ndiwa: Contributed reagents, materials, analysis tools or data; Wrote the paper.

### Funding statement

CKB, DE and NM were supported by the CGIAR system and in particular to the Livestock CRP http://www.cgiar.org/about582 us/our-funders). JOO was supported by the European Union's Horizon 2020 research and innovation programme under grant agreement No. 641918 through the AfricanBioServices Project as well as support from the German Research Foundation (DFG, Grant # 257734638).

### Data availability statement

Data associated with this study has been deposited at CG-Space under the data.ilri.org/ portal.

### Declaration of interests statement

The authors declare no conflict of interest.

### Additional information

No additional information is available for this paper.

## Figures and Tables

**Table A.1:** IMPACT model projections for the business-as-usual, sustainable development and Kenya vision 2020 scenarios.

		Business as Usual	Business as Usual	Sustainable Development	Kenya V2030
		2010	2030		
MODEL INPUTS					
Human Population	millions	40.51	60.59	57.97	45.55
GDP total	billion USD, 2005 prices	60.01	180.06	199.31	404.64
GDP per person	000 USD per person, 2005 prices	1.48	2.97	3.44	8.88
MODEL OUTPUTS					
Total production of beef	000 MT	460.75	780.79	789.10	1002.88
Total production of lamb	000 MT	92.21	154.24	155.91	184.81
Total production of Milk	000 MT	3618.77	5392.99	5427.80	8994.53
Slaughtered beef animals	000 head	2817.87	3375.13	3411.07	3372.50
Slaughtered lamb/mutton animals	000 head	7444.96	9273.85	9373.84	9270.76
Milked animals	000 head	10386.04	12781.76	12864.25	12732.82
Total net imports of beef	000 MT	0.00	53.51	46.75	0.00
Total net imports of lamb/muton	000 MT	0.00	1.46	0.00	0.00
Total net imports of milk	000 MT	0.00	0.00	0.00	0.00
Total net exports of beef	000 MT	9.06	0.00	0.00	51.20
Total lamb/mutton net exports	000 MT	5.73	0.00	0.82	11.41
Total net exports of milk	000 MT	61.21	191.70	422.31	4685.47
Total beef demand for household use	000 MT	451.69	834.30	835.85	951.68
Total lamb demand for household consumption	000 MT	86.48	155.70	155.09	173.39
Total milk demand for household use	000 MT	3259.85	4707.59	4508.62	3661.39

Source: Authors, using IMPACT model (Robinson et al., 2015).

## Appendix A Supplementary data

Table S1 and S2_V2.xlsx Table S1 and S2_V2.xlsx
